# Selection of optimal human myoblasts based on patient related factors influencing proliferation and differentiation capacity

**DOI:** 10.1038/s41598-025-96108-1

**Published:** 2025-04-05

**Authors:** Moritz Englich, Andreas Arkudas, Lilly Mengen, Raymund E. Horch, Aijia Cai

**Affiliations:** Department of Plastic and Hand Surgery, Laboratory for Tissue Engineering and Regenerative Medicine, University Hospital of Erlangen, Friedrich-Alexander University of Erlangen-Nürnberg (FAU), Krankenhausstr. 12, 91054 Erlangen, Germany

**Keywords:** Myoblasts, Myogenic differentiation, Myoblast proliferation, Multiple linear regression, Skeletal muscle, Tissue engineering, Stem-cell research, Translational research, Muscle stem cells

## Abstract

Human myoblasts (hMb) are a promising source for engineering skeletal muscle tissue. But sample-specific variabilities make research with human cells challenging. For the purpose of selecting hMb with adequate proliferation and differentiation properties, the influence of various patient related factors, including age, gender, BMI, anatomical sampling site and previous radio-/chemotherapy on hMb behavior was investigated in this study. Immunofluorescence staining and proliferation periods were analysed for proliferation capacity, while creatine kinase and cell viability assay, immunofluorescence staining and PCR were used to determine differentiation capacity. Using desmin expression, a multiple linear regression (MLR) model was established based on the above-mentioned patient related factors. Higher age and BMI, female gender and chemotherapy had a negative impact on desmin expression. Muscle type specific differences could also be seen. Previous radiotherapy led to senescence of hMb in large parts. Differentiation was mainly influenced by gender in a time-dependent manner, as well as by the anatomical collecting site. We were able to demonstrate the importance of analyzing patient characteristics for the purpose of hMb isolation. Using MLR, these patient characteristics can be used to predict the proliferation capacity of hMb as a step further towards translational application of skeletal muscle engineering and regeneration.

**Supplementary Information:**

The online version contains supplementary material available at 10.1038/s41598-025-96108-1.

## Introduction

The treatment of volumetric tissue loss is still a major problem in reconstructive surgery. Skeletal muscle tissue engineering offers a valuable approach to treat functional defects in patients^1,2^. To date, human myoblasts (hMb) derived from satellite cells (SC) are the only sufficient primary cell source for skeletal muscle tissue engineering. SC are activated in case of injury to the skeletal muscle and migrate as myoblasts (myogenic progenitor cells) to the site of the injury, where they mature and fuse to form multinucleated myotubes^3,4^.

Those primary muscle precursor cells can be isolated from muscle tissue and cultivated and differentiated to myotubes. However, besides the low number of isolatable cells^4–6^, studies using primary hMb often produce highly variable results^7,8^. This might result from different factors of the cell donors like age, gender or muscle type which have an impact on hMb proliferation and differentiation capacity as other studies have shown for other cell types^9,10^. To minimize deviations, many research groups are turning to animal cell lines, such as the C2C12 mouse cell line or try to increase the number of cells by using human-induced pluripotent stem cells^11,12^. However, these cells are not suitable for clinical applications due to their low myogenic potential^13^ and inefficient transformation rates^14^, besides the still existing concerns regarding the cancer risk^15,16^. Up to this point, primary hMb seem the only source for skeletal muscle tissue engineering purposes, so those patient factors need to be characterized in terms of hMb quality.

For instance, age and gender appear to have an influence on the proliferative and differentiation capacity of hMb^17–21^. Another interesting question is whether the Body-Mass-Index (BMI) also has an influence on the proliferative capacity of hMb, as has already been shown for other cell types, such as adipose tissue-derived stem cells^22,23^. In addition to the patient characteristics, the anatomical collection site, i.e. the donor muscle, also seems to influence the number of SC. For example, it has been shown that a 2–4 times higher number of SC can be found in the soleus muscle than in the vastus lateralis muscle^24^ and there seem to be muscle type related differences in old patients^25^.

Prior chemotherapy might lead to a sharp decline in muscle mass and a decline of SC concentration^26,27^. Similarly, radiotherapy is associated with a lower proliferation and differentiation capacity in vitro^28^. Therefore, the influence of prior treatments needs to be analyzed in order to assess whether the harvesting of cells for subsequent tissue engineering is advisable in this patient group.

Overall, the selection of suitable hMb for tissue engineering remains a major problem. In several studies, influencing factors have already been examined as described above. However, the results also suggest that focusing solely on individual patient features does not reflect reality, as patient characteristics and their influence on proliferation or differentiation behavior need to be considered simultaneously. Furthermore, we were interested in defining exclusion criteria for hMb to minimize setbacks due to an insufficient hMb proliferation or differentiation.

## Materials and methods

### Selection of study population and isolation

**Fig. 1:**
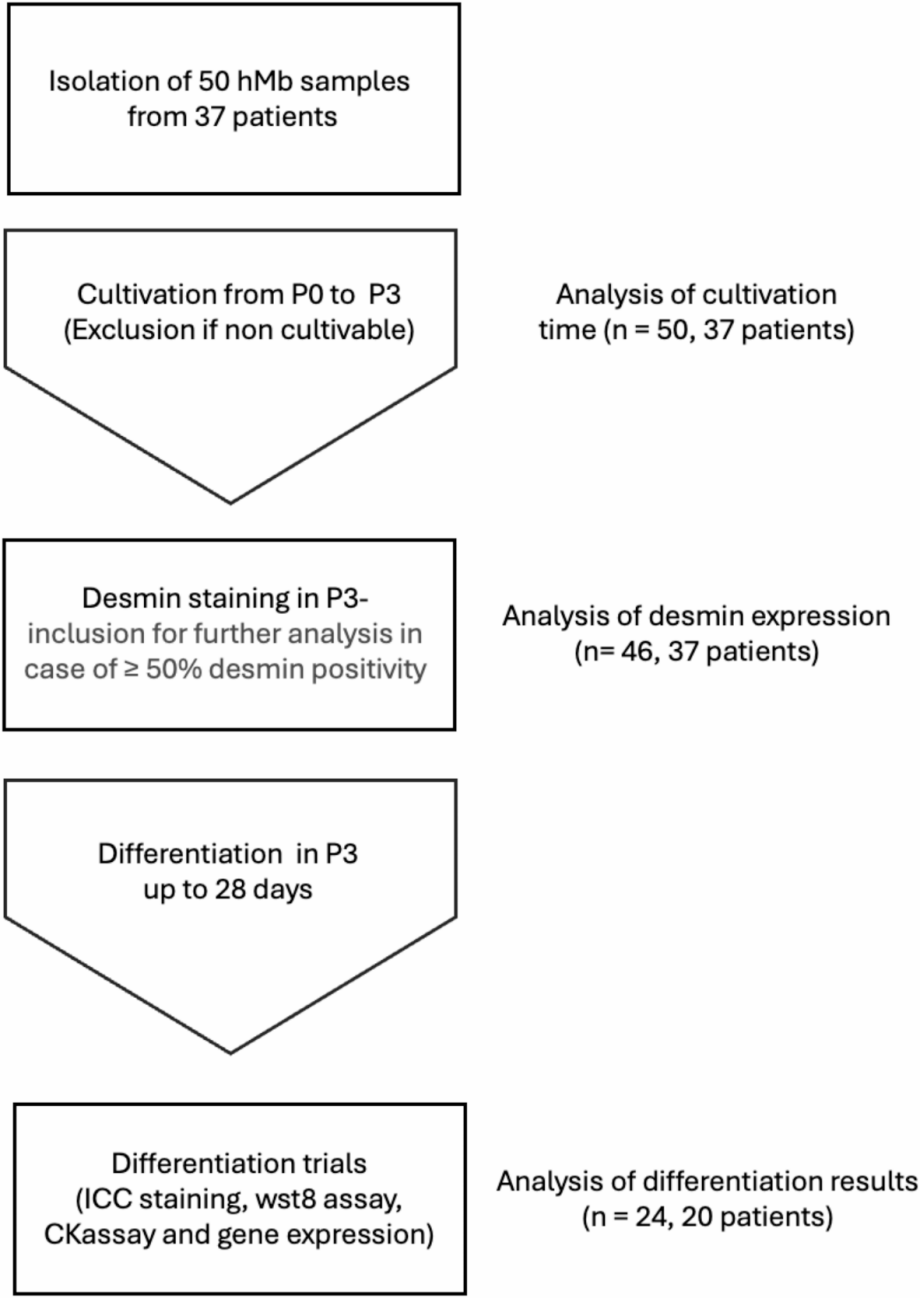
Overview of the study design.

The study was approved by the ethics committee of the University of Erlangen-Nürnberg (approval number: 424_18 B). Informed consent was obtained from all subjects involved in the study and all research was performed in accordance with the institutional guidelines and the Declaration of Helsinki. A total of 50 muscle samples were taken from 37 patients who underwent reconstructive surgery with free muscle flaps. In some cases of breast reconstruction, we were able to obtain tissue from both the defect site (pectoralis major muscle (PM)) and the donor site (rectus abdominis (RA) or gracilis muscle). Furthermore, in a patient who underwent bilateral breast reconstruction using deep inferior epigastric perforator (DIEP) flaps, we were able to take a sample from RA muscle as well as 2 additional samples, from the left unirradiated and from the right irradiated PM. For the isolation, we processed up to 5 g of skeletal muscle tissue according to a well-established protocol^29,30^. In short, after mechanical dissection, we placed the minced tissue in a collagenase II (CLS2, Biochrom GmbH, Berlin, Germany), dispase II (D4693-1G, Sigma) and trypsin (Sigma Aldrich) solution to digest the surrounding tissue. We then used a pre-plate technique, as described before^30,31^, using collagen-I-coated flasks (collagen type I from rat tail, C3867-1VL, Sigma). When cells reached a confluency of 70–80%, they were passaged five times (PP3-P1 - PP3-P5), by transferring them into new flasks. For every passage, the same concentration of living cells (2.5 x $\:{10}^{6}$ cells per T75 flask) was used. The third pre-plate (PP3-P3) was used for further differentiation experiments in case the desmin expression was ≥ 50% (Fig. 1). Myogenic differentiation was initiated by serum deprivation as described before^32^.

Groups were formed according to age, gender, BMI, muscle type and prior chemo- or radiotherapy to analyse the proliferation time as well as the degree of myogenic differentiation (Table 1). All isolations were successfully performed and we could include all samples in the proliferation trials.

**Table 1:** Overview of the groups for analysis of the proliferation time and the myogenic differentiation, using a mixed-effect model.

Examined factors	Proliferation time		Myogenic differentiation	
	Study population (*n* = 37)	Collected samples (*n* = 50)	Study population (*n* = 20)	Collected samples (*n* = 24)
Gender				
Male	12 (32.4%)	12 (24%)	7 (35%)	7 (29.7%)
Female	25 (67.6%)	38 (76%)	13 (65%)	17 (70.8%)
Age (years)				
< 50	10 (27%)	15 (30%)	7 (35%)	9 (37.5%)
≥ 50	27 (73%)	35 (70%)	13 (65%)	15 (62.5%)
BMI (kg/m^2^)				
< 25	11 (29.7%)	15 (30%)	5 (25%)	6 (25%)
≥ 25	26 (70.3%)	35 (70%)	15 (75%)	18 (75%)
Chemotherapy				
Yes	18 (48.6%)	26 (52%)	7 (35%)	9 (37.5%)
No	19 (51.4%)	24 (48%)	13 (65%)	15 (62.5%)
Radiation				
Yes	11 (29.7%)	11 (22%)	-	-
No	26 (70.3%)	39 (78%)	-	-
Skeletal muscle type				
Rectus abdominis	23 (62.2%)	23 (46%)	12 (57.1%)	12 (50%)
Pectoralis major	13 (35.1%)	14 (28%)	6 (28.6%)	6 (25%)
Gracilis	5 (13.5%)	5 (10%)	2 (9.5%)	2 (8.3%)
Vastus lateralis	4 (10.8%)	4 (8%)	2 (9.5%)	2 (8.3%)
Latissimus dorsi	2 (5.4%)	2 (4%)	1 (4.8%)	1 (4.2%)
Teres major	1 (2.7%)	1 (2%)	-	-
Soleus	1 (2.7%)	1 (2%)	1 (4.8%)	1 (4.2%)

The percentages regarding the skeletal muscle type do not add up to 100%, because in some cases, two muscle types from the same patient were used. Due to the low number of some muscle types, only samples from RA muscle and the PM muscle were used for differentiation analysis.

### Immunofluorescent staining

Desmin expression was determined for hMB in PP3-P1 to P5. As described before^29^, cells were incubated with desmin primary antibody (ab32362, Abcam) at a dilution of 1:50 and Alexa Flour (AF) 594 secondary antibody (A11012, Invitrogen) in a dilution of 1:500. HMb in PP3-P3 were used for staining against myosin heavy chain (MHC) and alpha-actinin. After incubation with a primary MHC (ab91506, Abcam) and alpha-actinin antibody (ab9465, Abcam) in a dilution of 1:100, AF 594 (A11012, Invitrogen) and AF 594 (A21125, Invitrogen) served as secondary antibodies at a dilution of 1:200. To stain the cell nuclei, diamidine-phenylindole-dihydrochloride (DAPI, Thermofisher Scientific Inc., USA) was used. Human fibroblasts served as negative control, while for every sample, a monostaining with secondary antibodies were performed. The cells were photographed at 3 predetermined positions with an Olympus IX81 (Olympus, Hamburg, Germany) under 10x magnification and then recomposed with the cellSens Dimension V.1.5 software. The number of cell nuclei was automatically analyzed using the software ImageJ (Version 1.53e, NIH, Bethesda, Maryland, USA) and the number of desmin-positive cells was set manually in relation to cells without desmin expression. In MHC and alpha-actinin stainings, the number of cell nuclei incorporated in myotubes in relation to the number of total cell nuclei was used to obtain the MHC fusion index (MFI) and the alpha-actinin fusion index (AFI)^33^. According to Jones et al. (2018), a myotube is defined as an elongated structure containing three or more nuclei within a single membrane. For the alpha-Actinin maturation index (AMI) and the MHC maturation index (MMI), the number of myotubes that have incorporated ≥ 5 nuclei was set in relation to the total number of myotubes^33^.

### Creatine kinase assay and cell viability assay

Cell Viability and Creatine Kinase (CK) activity were measured after 3, 7, and 14 days of myogenic differentiation using a wst8-assay kit (Colorimetric Cell Viability Kit (CCVK) I (WST-8)) and a Creatine Kinase Assay (Ab155901 Creatine Kinase Activity Assay Kit (Colorimetric)), as described before^34^. For the wst-8 assay, the provided protocol was used, and a negative control was performed by adding the CCVK-I solution to the same amount of proliferation medium without cells. For the CK assay, the enzyme activity was determined in a photometer at 450 nm through the amount of NADH generated by CK. A negative control, provided by the company, was used.

### Gene expression

RNA was isolated with RNeasy Mini Kit (Qiagen GmbH, Hamburg) and then transcribed into cDNA with Reverse Transcriptase Kit (QuantiTect, Qiagen GmbH) as described before^30^. Gene expression of key markers for myogenic maturation was measured using primers against MYOG (myogenin, a crucial regulator of myoblast differentiation and fusion) (forward primer: TGCCATCCAGTACATCGAGC, reverse primer: TGTGAGAGCTGCATTCGCTG), MYH2 (myosin heavy chain 2, an indicator of development of the contractile apparatus in differentiated myotubes) (forward primer: GGGCCTTTCAAGAGGGACAC, reverse primer: TGCGCTCCCTTTCAGACTTT), and ACTA1 (skeletal alpha actin 1, representing skeletal muscle-specific cytoskeletal organization) (forward primer: CACAATGTGCGACGAAGACG, reverse primer: CTCTCTTGCTCTGAGCCTCG). RPL13a (Ribosomal protein L13a) (forward primer: AAGTACCAGGCAGTGACAGC, reverse primer: TTCTCCACGTTCTTCTCGGC) was used as housekeeping gene. SsoAdvanced Universal SYBR Green PCR Supermix (Bio-Rad, Hercules, CA, USA) and a Light Cycler (Bio-Rad CFX96 TouchTM) were used for qPCR. The target genes were set in relation to the housekeeping gene RPL13a, using the $\:{2}^{-{\Delta\:}CT}$ method. A negative control was performed using RNAse free water only.

### Statistical analysis

All data are expressed as mean with standard deviations. Data normality was tested with Shapiro-Wilk test. Desmin expression was analysed via multiple linear regression (MLR). For cases where sample size was small due to exclusions related to low Desmin expression, analysis of variance (ANOVA) was used to compare groups. One-way ANOVA with Tukey’s test for multiple comparisons was used to analyse the passage time. Correlation between desmin expression and passage time was determined using Spearman correlation after testing for normal distribution using D’Agostino & Pearson test. Two-way ANOVA with Tukey’s test for multiple comparisons was used to compare the patient factors regarding the differentiation and passage time. A mixed-effect model was applied in case of missing values. Paired comparisons at the time points were carried out using repeated measures ANOVA with Tukey’s multiple comparisons test for post hoc analysis. Statistical analysis was performed using GraphPad Prism version 10.3 (La Jolla, CA, USA). A *p*-value ≤ 0.05 was considered statistically significant.

## Results

### ICC: desmin-expression

Desmin expression varied from 0 to 100% with a mean of 55.72% ($\:\pm\:$11.31). A MLR analysis was performed to analyze the influencing variables. To predict the expected desmin expression rate in P3 as accurately as possible, the MLR analysis included not only age, gender, BMI and muscle type, but also the presence of previous radiotherapy and chemotherapy (Table 2). Other influencing factors, such as the number of chronic illnesses like diabetes mellitus type II or arterial hypertension, were also taken into consideration, but could not contribute to clarifying the variance (ST 1). The factor “irradiation”, which also has no significance, was also analysed for all other questions and therefore left in the model for better transparency and completeness.

**Table 2:** Dependent factors used to build the MLR, either as categorical variable or as continuous variable (Age and BMI).

Examined factors	Study population (*n* = 37)	Collected samples (*n* = 46)
Gender		
Male	12 (32.4%)	12 (26.1%)
Female	25 (67.6%)	34 (73.9%)
Age		
(continuous variable, mean ± SD)		54.11 ± 11.31
BMI		
(contiunous variable, mean ± SD)		26.45 ± 4.32
Chemotherapy		
Received	18 (48.6%)	24 (52.2%)
Not received	19 (51.4%)	22 (47.8%)
Radiation		
Irradiated muscle	8 (21.6%)	8 (17.4%)
Non-irratiated muscle	29 (78.4%)	38 (82.6%)
Skeletal muscle type		
Rectus abdominis	22 (59.5%)	22 (47.8%)
Pectoralis major	11 (29.7%)	11 (23.9%)
Gracilis	5 (13.5%)	5 (10.9%)
Vastus lateralis	4 (10.8%)	4 (8.7%)
Latissimus dorsi	2 (5.4%)	2 (4.3%)
Teres major	1 (2.7%)	1 (2.2%)
Soleus	1 (2.7%)	1 (2.2%)

### Multiple linear regression equation to predict desmin expression

$$\begin{gathered} y = \beta _{0} + ~\beta _{1} x_{1} + \beta _{2} x_{2} + \beta _{3} x_{3} + \beta _{4} x_{4} + \beta _{5} x_{5} + \beta _{6} x_{6} + \beta _{7} x_{7} \hfill \\ \quad \quad + \beta _{8} x_{8} + \beta _{9} x_{9} + \beta _{{10}} x_{{10}} + \beta _{{11}} x_{{11}} + ~\varepsilon \hfill \\ \end{gathered}$$

where y is the dependent variable, $\:{x}_{i}$ are the independent variables, βi are the estimated regression coefficients of the used independent variables and *ε* is the model error.

A multiple R-squared value of 0.5983 and an adjusted multiple R-squared value of 0.4683 resulted from the regression equation using the above-mentioned influencing factors, which are defined as independent variables in Table 3. This means that the model can explain about 47% of the variation in the desmin expression of the isolated hMb. The variance of residuals was not constant across the predicted values (desmin expression in P3), so there was a moderate violation. The model can be accepted with an $\:{R}^{2}$ value of around 0.5, particularly as the factors included appear to have a significant influence on the predicted value. In addition, the analysis of variance appears to be significant, which shows that there is a linear correlation.

**Table:** 

Multiple *R*	*R* ^2^	Adjusted *R*^2^	Degrees of freedom	*P* value
0.7735	0.5983	0.4683	11	*p* = 0.0003

A negative linear relationship was found between desmin expression and increasing age (β1 = -1.822, *p* = 0.0002) as well as BMI (β3 = -3.187, *p* = 0.0128). In addition, gender also had a significant influence on desmin expression (*p* = 0.0069) as a higher desmin expression was found in hMb of male subjects (β2 = 40.54). Regarding the muscle type, hMb from the RA muscle showed higher desmin expression compared to hMb from the teres major (β6 = -109.7, *p* = 0.0015), gracilis (β8 = -40.72, *p* = 0.0140) and soleus muscle (β9 = − 86.40, *p* = 0.0132). In contrast, extractions from the PM muscle (β7 = -15.40, *p* = 0.1883), latissimus dorsi (β4 = -28.36, *p* = 0.2439) or vastus lateralis muscles (β5 = -30.62, *p* = 0.0958) did not differ significantly from the RA muscle group. While there was no correlation between prior irradiation and desmin expression (β10 = 12.60, *p* = 0.3332), a strong negative influence of chemotherapy on the desmin expression was found (β11 = -40.20, *p* = 0.0001). Strong multicollinearity, for example due to a specific muscle type and simultaneous irradiation, was excluded by determining the variance inflation factor (VIF) (Table 3), which was below 5 and close to 1 for each parameter.

**Table 3:** Shows all used parameters, which were used to carry out the MLR analysis.

Parameter	Variable	Estimate	95% CI (asymptotic)	*P* value	VIF
β0	Intercept	223.3	146.2 to 300.4	< 0.0001	1,363
β1	Age	-1.822	-2.709 to -0.9353	**0**.**0002**	2,193
β2	Gender [male]	40.54	11.88 to 69.20	**0**.**0069**	1,535
β3	BMI	-3.187	-5.652 to -0.7212	**0**.**0128**	1,36
β4	Muscle type [Latissimus dorsi]	-28.36	-76.97 to 20.24	0.2439	1,451
β5	Muscle type [Vastus lateralis]	-30.62	-66.95 to 5.705	0.0958	1,23
β6	Muscle type [Teres major]	-109.7	-174.3 to -45.02	**0**.**0015**	1,279
β7	Muscle type [Pectoralis major]	-15.4	-38.70 to 7.909	0.1883	1,367
β8	Muscle type [Gracilis]	-40.72	-72.65 to -8.794	**0**.**014**	1,327
β9	Muscle type [Soleus]	-86.4	-153.5 to -19.26	**0**.**0132**	1,169
β10	Irradiation [yes]	12.6	-13.48 to 38.64	0.3332	1,247
β11	Chemotherapy [received]	-40.2	-21.20 to -59.19	**0**.**0001**	1,363

* *p* < 0.05, ** *p* < 0.01, *** *p* < 0.001.

### Proliferation time

In addition to the desmin expression, the proliferation periods were determined, i.e. the time required for a predefined cell concentration (3.0-3.5 × 10^4^ cells per 1 cm², this equals 2.3–2.6 × 10^6^ cells per T75 flask) to reach 70–80% confluence in a T75 culture flask. The periods were determined for passages 0 to 5 (P0 - P5). Cultivation time from P0 (i.e. the myoblast suspension after pre-plating) to P1 took significantly longer than in the subsequent passages (P0-> P1 (*n* = 49) vs. P1-> P2 (*n* = 47): *p* = 0.0120, vs. P2-> P3 (*n* = 44): *p* = 0.0124, vs. P3-> P4 (*n* = 38): *p* = 0.0110 and vs. P4-> P5 (*n* = 25): *p* = 0.0016).

**Fig. 2:**
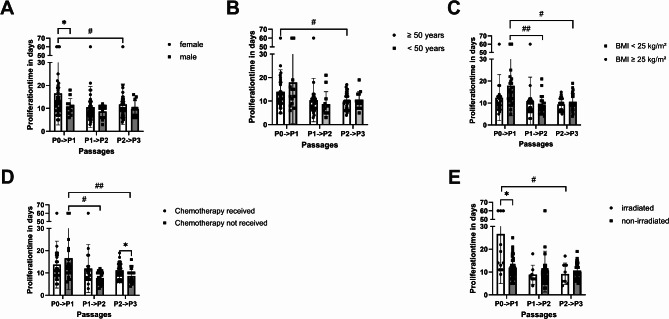
Mixed-effect model of the proliferation periods from P0 to P3. * *p* < 0.05, ** *p* < 0.01, *** *p* < 0.001 (Mixed-effect analysis with Tukey’s correction for multiple comparisons).

To analyse the effect of the patient characteristics, a mixed-effect model was set up, because some hMb isolations could not be cultivated across all passages (Fig. 2). It considers the patient factors and the passage number. While age and gender show no influence on proliferation time, radiotherapy shows a strong influence (*p* = 0.0032). Chemotherapy (*p* = 0.0158) and BMI (*p* = 0.016) show a time-dependent influence. Prior irradiated hMb and those from female subjects show significantly higher passage times from P0 to P1 (female vs. male: *p* = 0.024, irradiated vs. non-irradiated: *p* = 0.0491).

The negative influence of irradiation can be clearly seen by directly comparing the light microscopic images of the hMb isolations of an irradiated and a non-irradiated pectoralis major muscle from the same patient (SF 1). The hMb from the irradiated pectoralis major muscle show clear signs of senescence, which is presumably due to the previous irradiation.

Desmin expression (in P3), a well-established proliferation marker, was checked for correlation with the passage times (from P0 to P3), as a potential new proliferation marker. As some data sets were not normally distributed (desmin expression: *p* = 0.0177, P0-> P1: *p* < 0.0001, P1-> P2: *p* < 0.0001, P2-> P3: *p* = 0.3796), the non-parametric Spearman correlation was used. All r coefficients for correlation between desmin expression and proliferation time from P0-> P1, P1-> P2 and P2-> P3 were around 0, so no direct correlation between the desmin expression and the passage time was found (ST 2).

### ICC: MHC- and alpha-actinin expression

MHC and alpha-actinin expressions, which serve as late myogenic differentiation markers, were determined in form of the fusion index (MFI, AFI) and the maturation index (MMI, AMI). Examples from the ICC staining can be found in the supplemental materials (SF 2). The influence of the various patient characteristics on myogenic differentiation is described below.

#### Gender

**Fig. 3:**
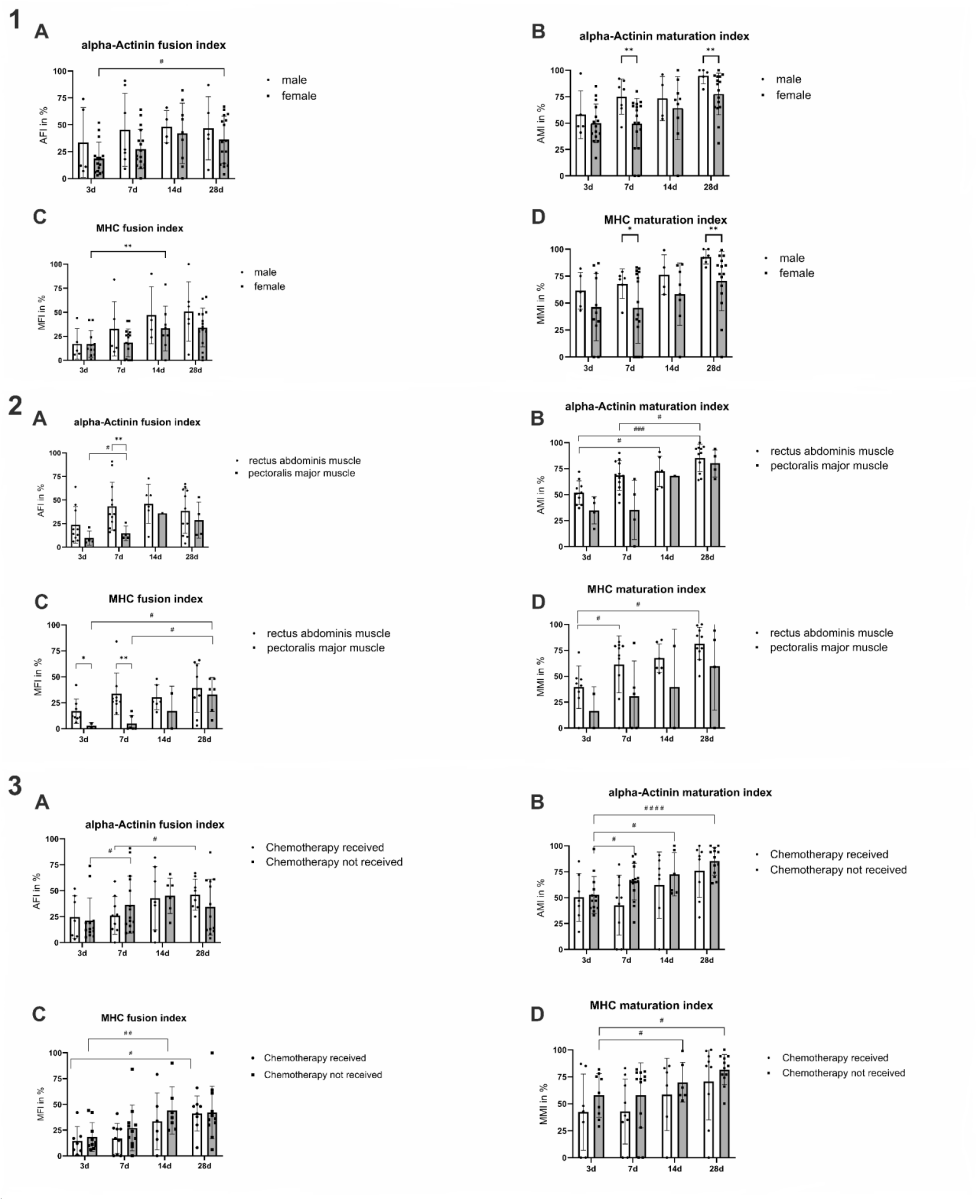
**1** shows the influence of gender and time on alpha-actinin (**A**, ** B**) and MHC expression (**C**, ** D**). In addition to significant differences within the groups, there are higher maturation indices at day 7 and 28 in the male group (**B**, ** D**). **2** shows muscle type specific differences between the PM muscle and the RA muscle. Higher fusion indices are seen on day 3 for MHC (**C**) and day 7 for alpha-actinin and MHC (**A**, **C**) in hMb isolations from RA muscle. The maturation indices increased from day 3 to 28 for RA muscle (**B**, ** D**), while samples from PM showed an increase in fusion indices (**A**,** C**). **3** The influence of chemotherapy and differentiation time on alpha-actinin and MHC expression is shown. No difference was found between subjects who had received chemotherapy and those who had not. There were significant increases for all measured indices (**A**, **B**, **C** and **D**) in both groups. * *p* < 0.05, ** *p* < 0.01, *** *p* < 0.001 (Mixed-effect analysis with Tukey’s correction for multiple comparisons).

Gender had no influence on alpha-Actinin expression (AFI: *p* = 0.0886, AMI: *p* = 0.0757) or the MFI (*p* = 0.1094), but on the MMI (*p* = 0.0498). The post-hoc analysis showed that the AMI and MMI were significantly higher at day 7 (AMI: *p* = 0.0088, MMI: *p* = 0.0378) and 28 (AMI: *p* = 0.0099, MMI: *p* = 0.0091) in males than in females (Fig. 3(1)).

#### Age and BMI

Age and BMI did not show an influence on alpha-actinin or MHC expression (Age: AFI: *p* = 0.2118, AMI: *p* = 0.3136, MFI: *p* = 0.6001, MMI: *p* = 0.8272; BMI: AFI: *p* = 0.8109, AMI: *p* = 0.6312, MFI: *p* = 0.9368, MMI: *p* = 0.9495 ). Furthermore, the post-hoc analysis could not detect any intergroup differences.

#### Differences between the rectus abdominis and the pectoralis major muscle

HMb isolated from the RA muscle were compared with those from the PM muscle (Fig. 3(2)). Two PM muscle samples were excluded from the analysis to rule out a possible influence of irradiation. No general influence of the muscle type on the MMI (*p* = 0.0736) or AFI (*p* = 0.1104) could be determined, at least between hMb from these two muscle types. Nevertheless, the post-hoc analysis revealed a significantly higher AFI on day 7 in the RA than in the PM muscle (*p* = 0.0039). In contrast to the AFI and MMI, the mixed-effect model shows an influence on the AMI (*p* = 0.0499) and MFI (muscle type: *p* = 0.0155). In addition, there was a significantly higher MFI on day 3 (*p* = 0.0169) and 7 (*p* = 0.0026) in the RA muscle group than in the PM muscle group. Due to an insufficient samples size, PM could not be analyzed for the AFI and AMI on day 14.

#### Chemotherapy

A significant influence of chemotherapy on alpha-actinin or MHC expression could not be seen (Fig. 3(3)) (AFI: *p* = 0.9823, AMI: *p* = 0.1304, MFI: *p* = 0.3041, MMI: *p* = 0.1522), while all indices increased over time (AFI: *p* = 0.0076, AMI: *p* < 0.0001, MFI: *p* = 0.0006, MMI: *p* = 0.0116).

### Cell viability

**Fig. 4:**
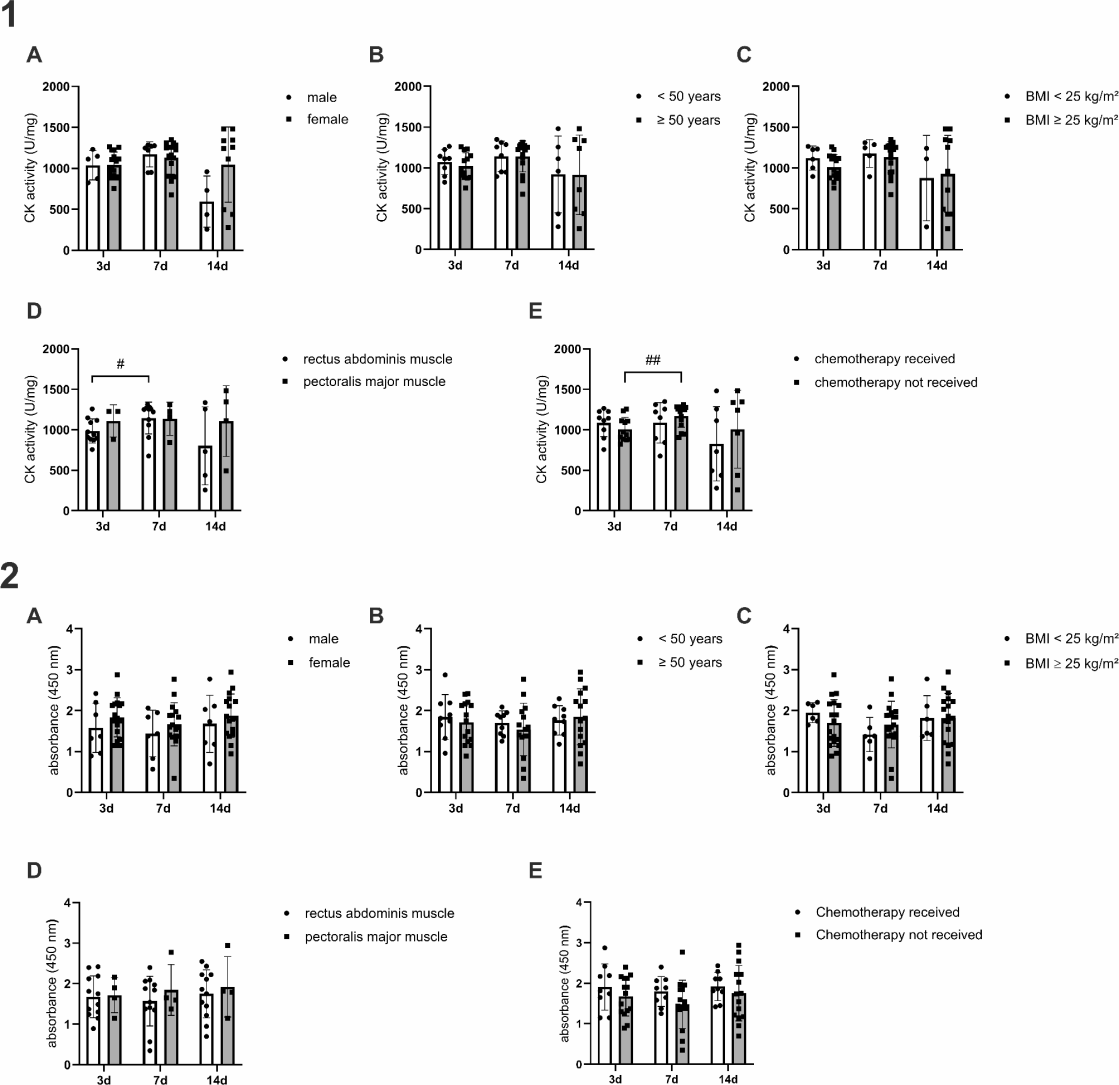
**1** shows the CK activity of differentiated hMb determined with CK activity assay. No direct influence of time and gender (**A**), age (**B**), BMI (**C**), muscle type (**D**) and chemotherapy (**E**) could be observed. CK activity increased within 3 to 7 days in RA and in the group without prior chemotherapy. CK activity in U/mg of protein in the samples is expressed as mean ± standard deviation. **2** shows the cell viability of differentiated human hMb determined with wst-8-assay. No influence of time and gender (**A**), age (**B**), BMI (**C**), muscle type (**D**) and chemotherapy (**E**) could be observed. Absorbance at a wavelength of 450 nm is expressed as mean ± standard deviation. # *p* < 0.05, ## *p* < 0.01 (repeated measures two-way ANOVA with Tukey’s multiple comparison test).

he two-way ANOVA (Fig. 4(2)) showed no significant influence of the factors or time on the cell viability (gender: *p* = 0.2138, age: *p* = 0.6898, BMI: *p* = 0.9879, muscle type: *p* = 0.5582 and chemotherapy: *p* = 0.1636). In the post-hoc analysis, cell viability did not differ between male and female subjects (3 days: *p* = 0.3408, 7 days: *p* = 0.3844 and 14 days: *p* = 0.5202), between < and ≥ 50 year-olds (3 days: *p* = 0.5569, 7 days: *p* = 0.4173 and 14 days: *p* = 0.6897), between those with a BMI of < and ≥ 25 kg/$\:{m}^{2}$(3 days: *p* = 0.1512, 7 days: *p* = 0. 2813 and 14 days: *p* = 0.9918), between hMbs from the RA or PM muscle (3 days: *p* = 0.8906, 7 days: *p* = 0.4796 and 14 days: *p* = 0.6936) or between subjects who received a chemotherapy and those who did not (3 days: *p* = 0.3250, 7 days: *p* = 0.1226 and 14 days: *p* = 0.4448).

### Creatine kinase activity

Gender showed no general influence (*p* = 0.1381), but a time-dependent influence on the CK activity (gender x time: *p* = 0.01539). In contrast, none of the other investigated factors, like age (*p* = 0.9257), BMI (*p* = 0.7816), muscle type (*p* = 0.1990) or chemotherapy (*p* = 0.5188) showed an influence in general or time-dependent changes. In the gender factor post-hoc analysis no group showed a significant higher CK activity on the measured days (3d: *p* = 0.9674, 7d: *p* = 0.5918, 14d: 0.06589). In the multiple comparison for the factors age and BMI, no significant differences were found between the < and ≥ 50-year-olds (3d: *p* = 0.4826, 7d: *p* = 0.9801, 14d: *p* = 0.9836) and between the normal-weight and overweight individuals (3d: *p* = 0.1524, 7d: *p* = 0.6180, 14d: *p* = 0.8884) on the days measured. Similarly, there were no significant differences between hMb from the RA and the PM muscle at day 3 (*p* = 0.3958), 7 (*p* = 0.9325) and 14 (*p* = 0.3533), nor any intergroup differences between individuals with chemotherapy and without on day 3 (*p* = 0.2808), 7 (*p* = 0.4012) or 14 (*p* = 0.4868). Overall, there was no difference in CK activity between the different subgroups (Fig. 4(1)).

### Gene expression

PCR was successfully performed in 18 samples from 18 patients (SF 3). The target genes ACTA1, MYH2 and MYOG were set in relation to the housekeeping gene RPL13a. The mixed-effects analysis (SF 4) showed a general influence of gender (*p* = 0.0017) and chemotherapy (*p* = 0.0092) on myogenic gene expression. Age and BMI showed no overall influence on the gene expression (age: *p* = 0.2293, BMI: *p* = 0.0631), but there seems to be a time related significant difference between the ≥ and < 50-years old (age x time: *p* = 0.0384). The extended post-hoc multiple comparison showed no significant differences in ACTA1, MYH2 or MYOG expression between the formed groups, so for gender and chemotherapy no group showed a constantly higher gene expression at the measured days. A comparison between the muscle types could not be performed due to insufficient n-numbers.

## Discussion

The results of the current study show various factors to be considered when it comes to hMb proliferation and differentiation capacity. Furthermore, for the selection of hMb for research or therapeutic purposes, both the proliferation-related and the differentiation-related factors must be considered. A unilateral explanation of individual influencing factors, as has been done in previous studies^17,20,24^, cannot be considered, as it seems that the combination of several factors has a substantial effect on myogenic proliferation or differentiation. Thus, it is essential to use forms of analysis that can take multiple variables into consideration at the same time.

To the best of our knowledge, we were able to establish the first MLR to predict desmin expression based on patient characteristics. This statistical method ensures that the relationship between multiple factors is accurately captured while avoiding monoline correlations between variables. Typical influencing factors such as age, BMI, and gender showed a strong influence. This should be considered when selecting suitable hMb for research purposes to avoid unnecessary or unpromising isolations.

Certain previous treatments had a strong negative influence on proliferation capacity, such as chemotherapy in the case of desmin expression or radiotherapy in the case of the cultivation time. These influencing factors should also be considered when selecting suitable subjects for further research projects and corresponding samples should be excluded, if possible, to avoid biases in the statistical analysis. Even if it appears that irradiation has no influence on desmin expression, but only on the proliferation time in low passages, it should be noted that the exclusion of some samples, for example due to non-growth ability, leads to biases in the subsequent examinations, as desmin expression was assessed in P3. The significantly longer passage times of irradiated hMb at P0/P1 are likely attributable to their reduced tolerance to isolation stress, including mechanical and thermal stress. This delay not only reflects the initial challenges faced by irradiated cells but also contributes to a longer overall time to reach later passages, which should be considered when selecting cells for future experiments. Notably, significant differences in passage times were observed primarily between P0 and P1, with later passages showing more stable trends.

The same applies to differentiation, where we were unable to investigate a possible influence in more detail, as only 2 irradiated samples of the original 11 samples were included in the differentiation experiments.

The anatomical sampling site also appears to have a significant influence on proliferation capacity. The individual muscles that showed a negative influence in the MLR should only be taken with caution due to the small n-number. However, it supports the previously published work that has shown a significant difference in the sampling site^24,25^. Therefore, in future studies using hMb, samples from the same muscle should be used where possible or analysed for intermuscular differences. Differences between the PM and RA muscle, which we analysed, were not found regarding proliferation capacity. Unfortunately, the other muscle types could not be included in the statistical analysis due to an insufficient n-number, which means that no reference can be made to previous studies that focused primarily on other muscle types^24,25^.

In addition to the influence on proliferation capacity in general, we took a closer look at patient-related influence on the proliferation time of hMb. It could be shown that the proliferation time between passages decreases with increasing passage time, but not significantly. Particularly from P0 to P1, long proliferation times are to be expected which could be due to the physical stress caused by the previous isolation. In these low passages, previous irradiation and female gender have a negative effect on the proliferation time. However, there appear to be strong individual influences on the samples, so that a generally valid model to predict the passage time could not be established, as was the case with desmin expression.

Furthermore, it was of interest whether the passage time is suitable as a proliferation marker. Since none of the passage times correlated with desmin expression, they cannot be used directly to predict proliferation ability. This means that samples should not be excluded from further analyses based on a high passage time. However, in the future, lower exclusion criteria for the cultivation time should be adopted than were used in the study with a cut-off value of 60 days. Except for the irradiated samples, which did not show sufficient growth even after 60 days, all other isolations had growth times with a maximum of 25 days, so that 30 days, for example, can certainly be considered as a cut-off value. Therefore, if considering not only the desmin expression, but also the duration of cultivation or cultivability, muscle samples that have previously been irradiated or samples from subjects who have previously undergone chemotherapy should be avoided.

To do justice to the strong variance of the desmin expression in P3 as we did to the strong variance of the proliferation periods, an inclusion limit of only 50% desmin expression was chosen. In previous studies^30^, we did not find such a strong variance and the isolations mostly showed higher desmin expression rates of around 95%, which could be explained by the lower n number and a more carefully selection of subjects, as the aim of these studies was not to record patient characteristics.

In general, it appears that the investigated patient-related factors have less of an effect on differentiation than they have on proliferation. The CK activity showed that the investigated factors have no influence on differentiation except for gender in a time-dependent manner, which could be of value for long-term differentiation. The male group showed no higher CK activity than the female group, which is in contrast to previous work, that focused on post damage and exercise response^35,36^. But regarding the time-dependent influence of gender, differences in CK activity are not ruled out during longer differentiation periods.

The ICC results for the intracellular filament expression show a more inhomogeneous picture than the results of the assay described above. While the gender-specific influence, like for the CK activity, could be confirmed, especially for the maturation indices, other factors like age, BMI and chemotherapy showed no influence here. However, a muscle-specific influence certainly appears to be present as hMb isolations from the RA showed a higher differentiation capacity compared to hMb from the PM. On a molecular level, it seems that only gender and prior chemotherapy influenced the gene expression of myogenic differentiation markers.

The study has several limitations. First, Desmin was our sole myoblast proliferation marker. However, it is a well-established myogenic intermediate filament protein, expressed early in myogenesis, and remains present throughout both the proliferation and differentiation stages of myoblasts^37–39^. Since it cannot be ruled out that some myoblasts may already be in the differentiation process despite careful suppression, desmin offers the most reliable marker for detecting a broad range of myoblasts, including those in various developmental stages, within the isolated cell cultures. The expression of other myogenic markers such as Pax7, MyoD and CD56^37,38,40^ are highly variable in different myogenesis stages of the cells. This complexity in marker expression may introduce interpretative challenges as distinct subpopulations within the isolated cell pool are marked. While Desmin expression alone may not capture the full spectrum of myogenic potential, it serves as a practical and widely accepted marker in the field as has been practiced in several other studies^41–48^. Another limitation is the small sample size, which may affect statistical power, particularly when comparing multiple factors simultaneously (e.g., in multiple linear regression analyses). This also impacted the differentiation analysis, as the limited proliferation of certain samples led to a decreased number of samples available for analysis. The high number of breast reconstructions led to heterogenous group distribution with women and PM and RA muscle samples predominating. This resulted in inhomogeneous subgroups and it cannot be ruled out that several factors influenced each other, for example the female gender and the PM muscle. Thus, the unexplained variance of just over 50% cannot necessarily be attributed only to inter-individual differences. In addition, it would have been highly desirable to carry out a MLR analysis for the differentiation results and the proliferation periods to be able to exclude mutually influencing factors as generously as possible. However, due to the high exclusion rate of over 50% of the samples (from the original *n* = 50 (37 patients) to *n* = 24 (20 patients)), no standardized model could be found.

## Conclusion

The study shows how diverse the factors influencing proliferation and differentiation capacity of hMb are and how important it is to consider them carefully before isolation for preclinical or clinical purposes. While patient characteristics seem to specifically influence hMb proliferation, myogenic differentiation seems to be less dependent on those parameters as far as can be evaluated based on limited sample size. When considering the proliferation in context with the differentiation results, hMb from subjects who have previously undergone radiotherapy or chemotherapy, should be avoided. In addition, depending on intraoperative possibilities, hMb from the RA muscle should be preferred over hMb from the PM muscle due to superior differentiation results. HMb from young male patients with a lower BMI without prior radiotherapy or chemotherapy have been shown to be particularly favorable for in-vitro proliferation while for differentiation, male gender seems to be the only relevant of the previously mentioned characteristics.

In addition, the proliferation time between passages was not suitable to establish a new proliferation marker due to excessive differences between passages and a lack of correlation with already established proliferation markers such as desmin expression.

## Electronic supplementary material

Below is the link to the electronic supplementary material.

Supplementary Material 1

## Data Availability

The datasets used and analysed during the current study are available from the corresponding author on reasonable request.
