# Supplementary material for: Selection of optimal human myoblasts based on patient related factors influencing proliferation and differentiation capacity
Source: Sci Rep. 2025 Apr 5;15:11714. doi: 10.1038/s41598-025-96108-1 (PMC11972305; doi:10.1038/s41598-025-96108-1)
Supplement: Supplementary file 1 — Supplementary Material 1 [file 41598_2025_96108_MOESM1_ESM.docx]

| Chronic illnesses | Study population (n = 37) | Collected samples (n = 46) |
| --- | --- | --- |
|  |  |  |
| *Chronic illnesses included* |  |  |
| (continous variable, mean ± SD) |  | 1.826 (± 1.338) |
|  |  |  |
| *Diabetes mellitus type II* |  |  |
| Yes | 5 (13.5%) | 5 (10.9%) |
| No | 32 (86.5%) | 41 (89.1%) |
|  |  |  |
| *Arterial Hypertension* |  |  |
| Yes | 10 (27%) | 10 (21.7%) |
| No | 27 (73%) | 36 (78.3%) |

**ST 1:** Frequency of secondary diseases in study population. The number of secondary diseases varied between 0 and 7.

| desmin expression vs. | Spearman r | 95% confidence interval | p value |
| --- | --- | --- | --- |
| P0->P1 | 0,03212 | -0,2688 to 0,3274 | 0,8322 |
| P1->P2 | -0,1788 | -0,4530 to 0,1263 | 0,2346 |
| P2->P3 | -0,06519 | -0,3566 to 0,2378 | 0,6669 |

**ST 2**: Correlation matrix using nonparametric Spearman correlation.


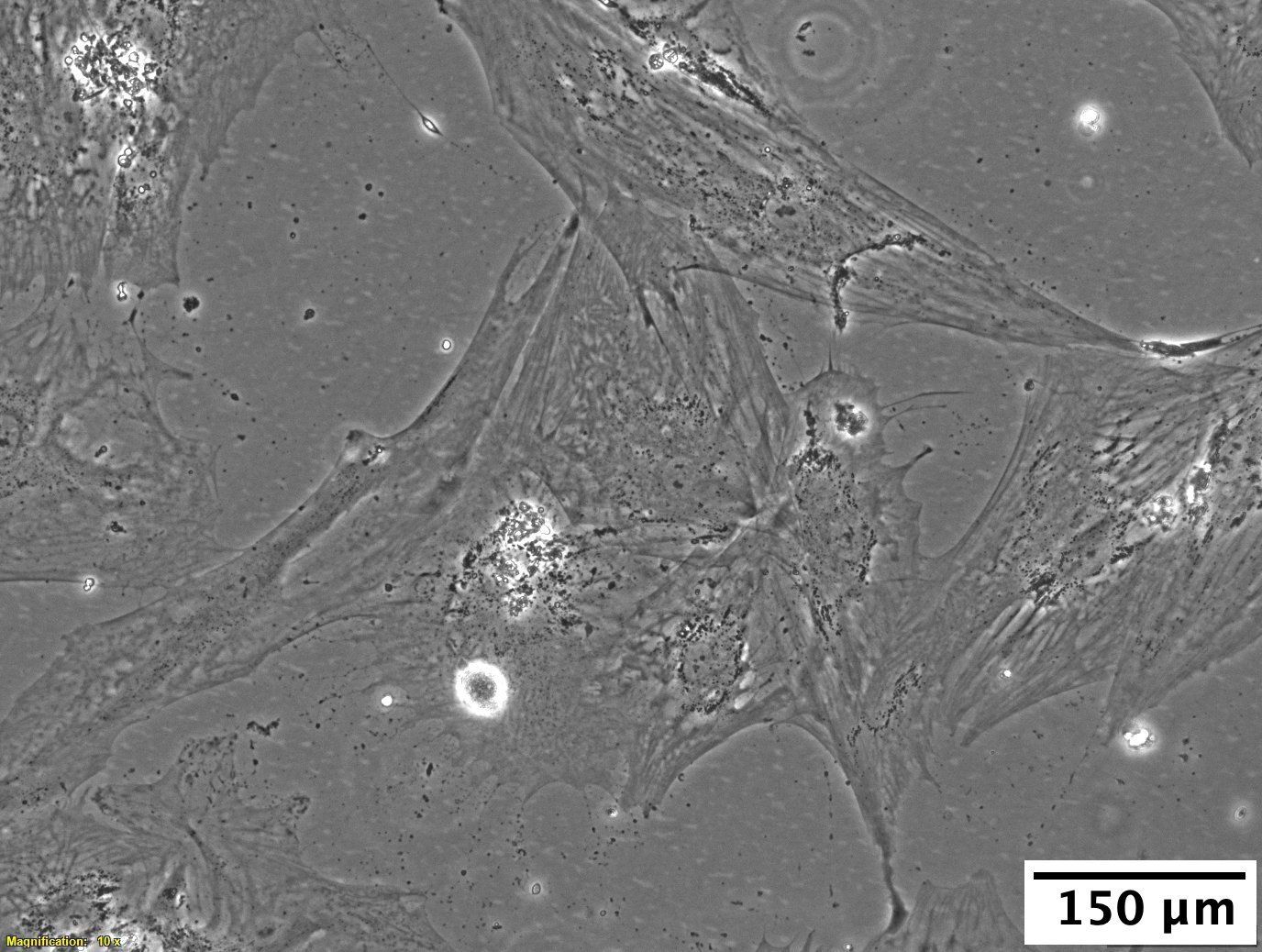

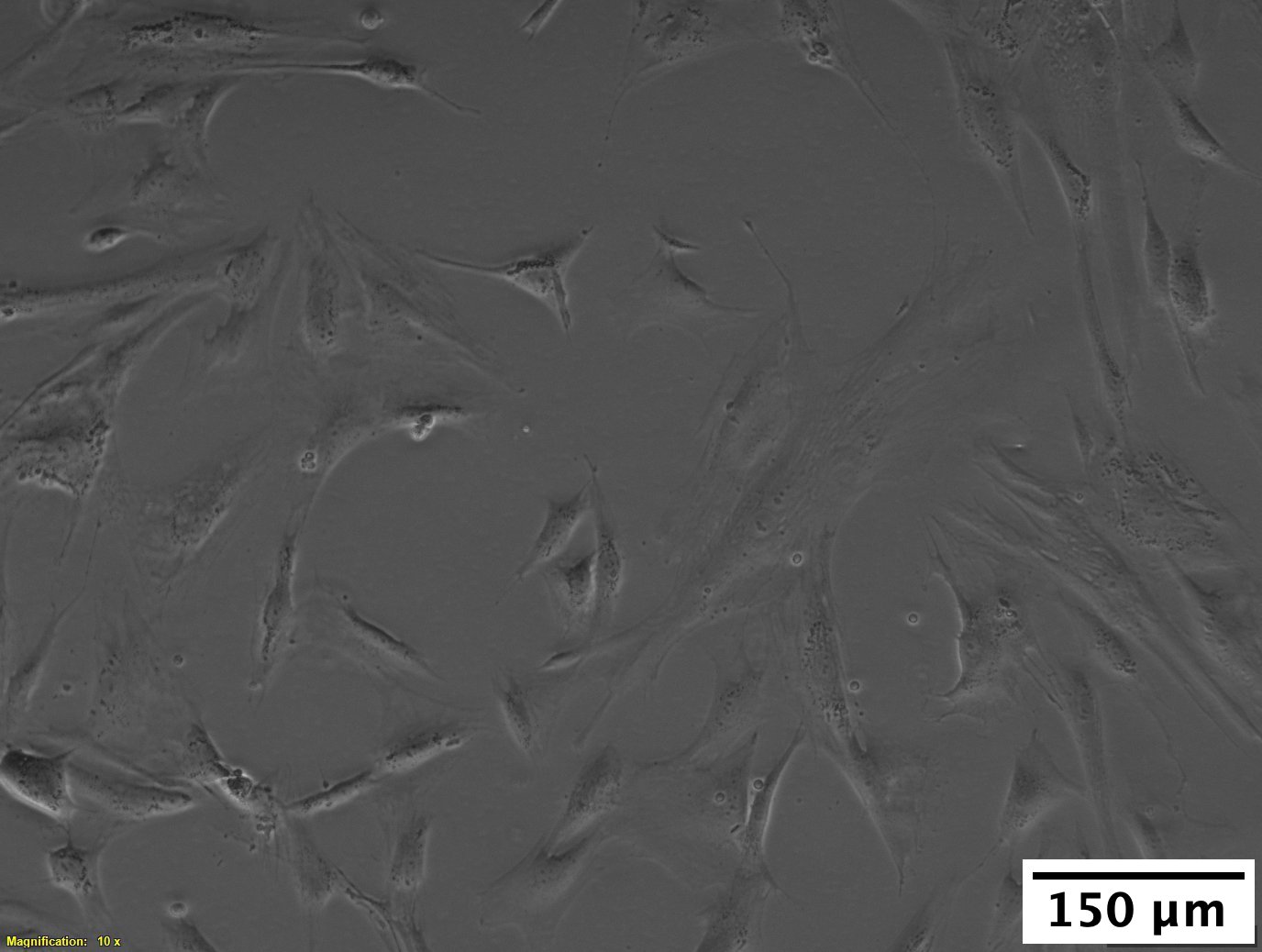


A

B

**SF 1:** Optical microscope image, each at 10x magnification, of two hMb isolations from the same patient who received a prophylactic mastectomy with no prior oder subsequent irradiation on one side (**A**) and was irradiated on the other side as part of breast cancer therapy (**B**). There are clear morphological deviations from the normal appearance of hMb that are compatible with senescence of the irradiated cells.


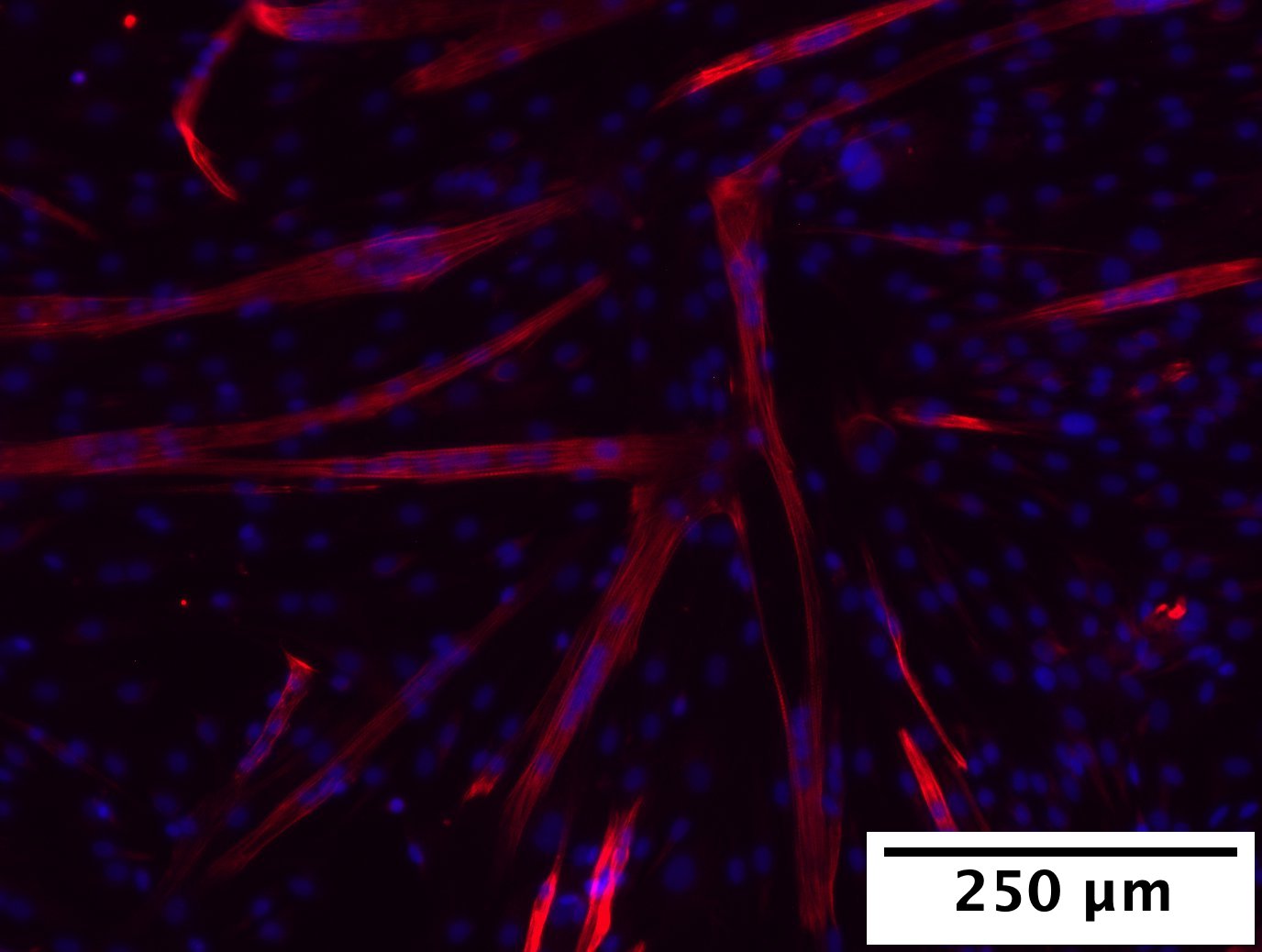

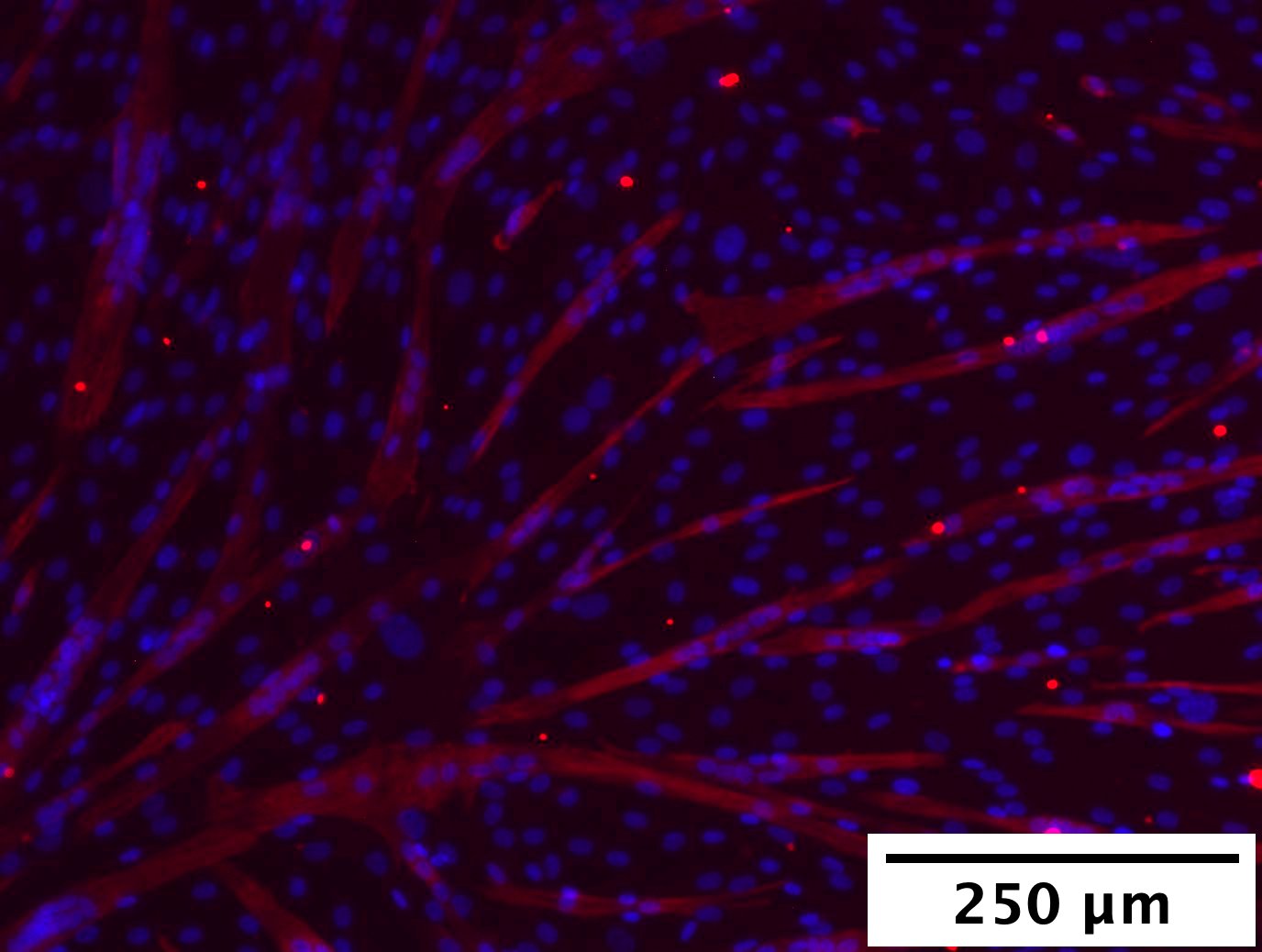

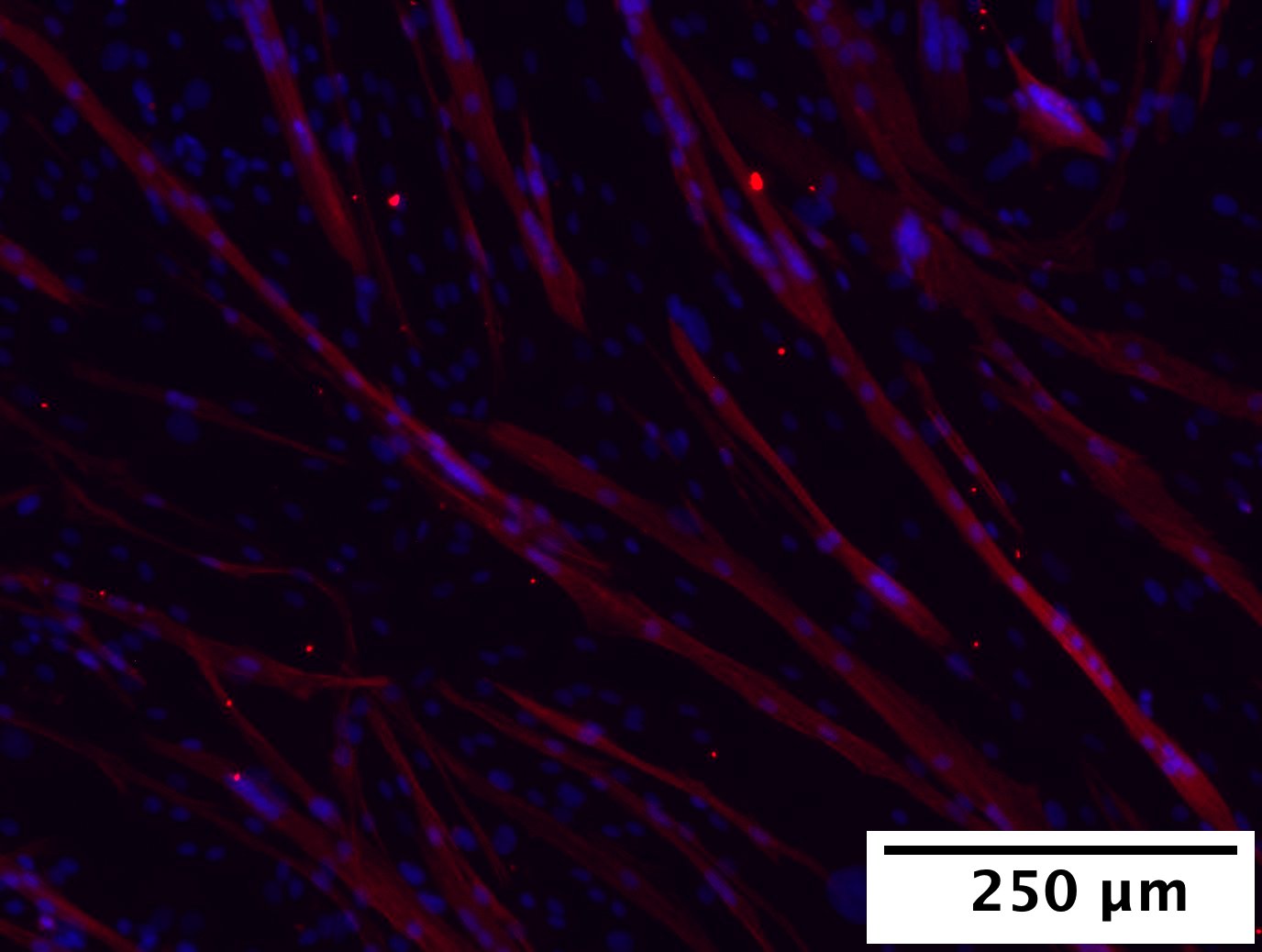

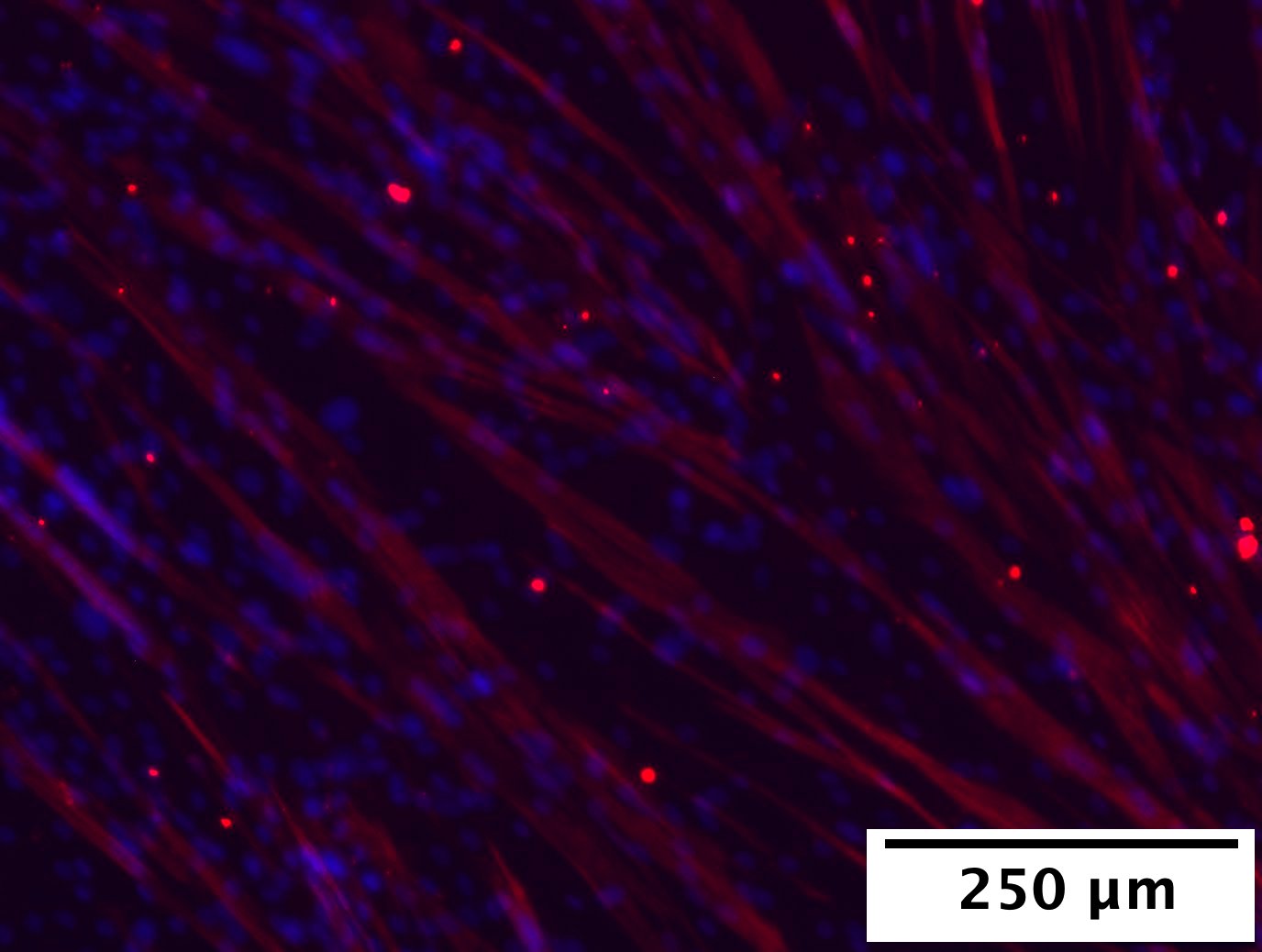

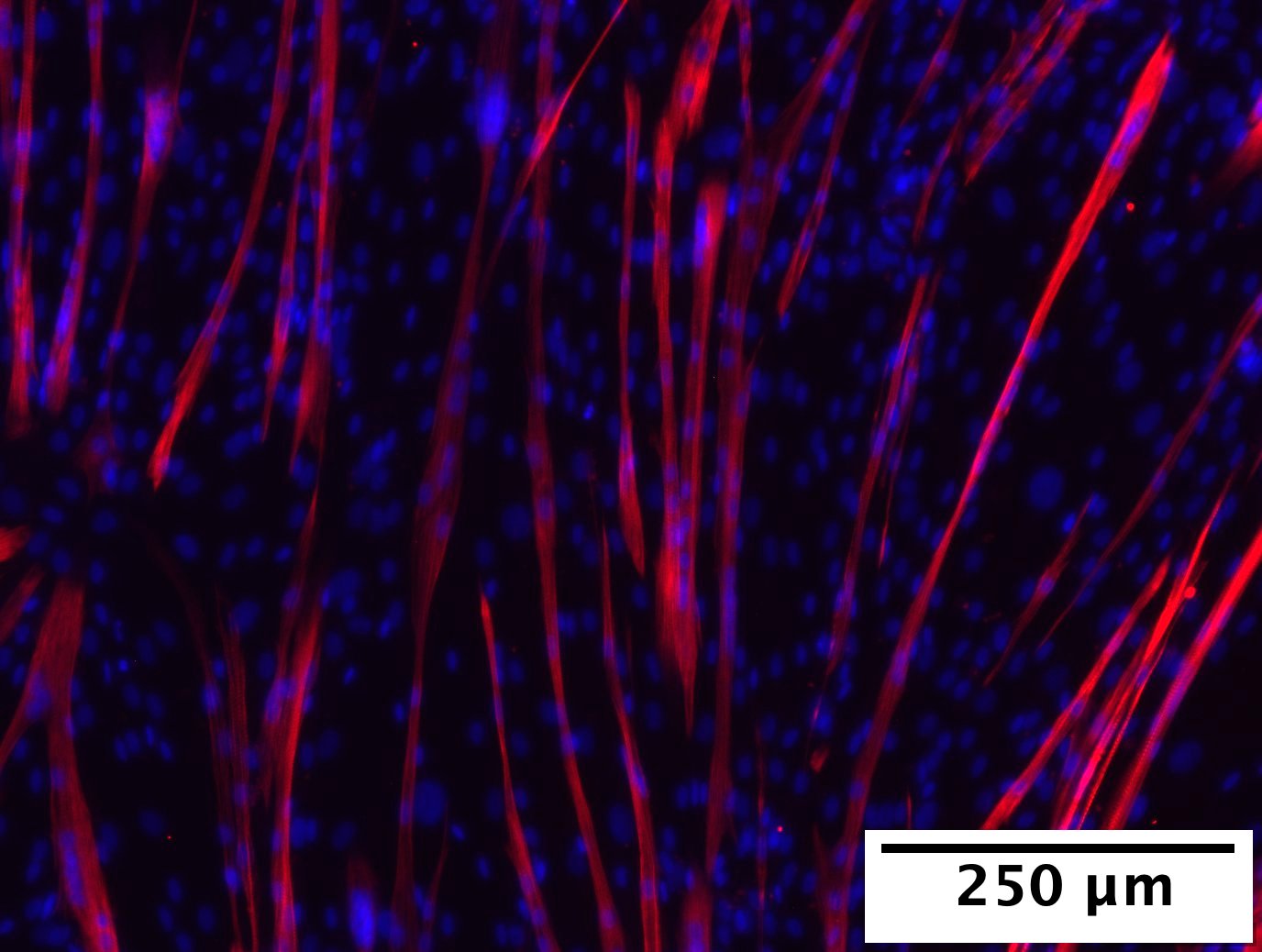

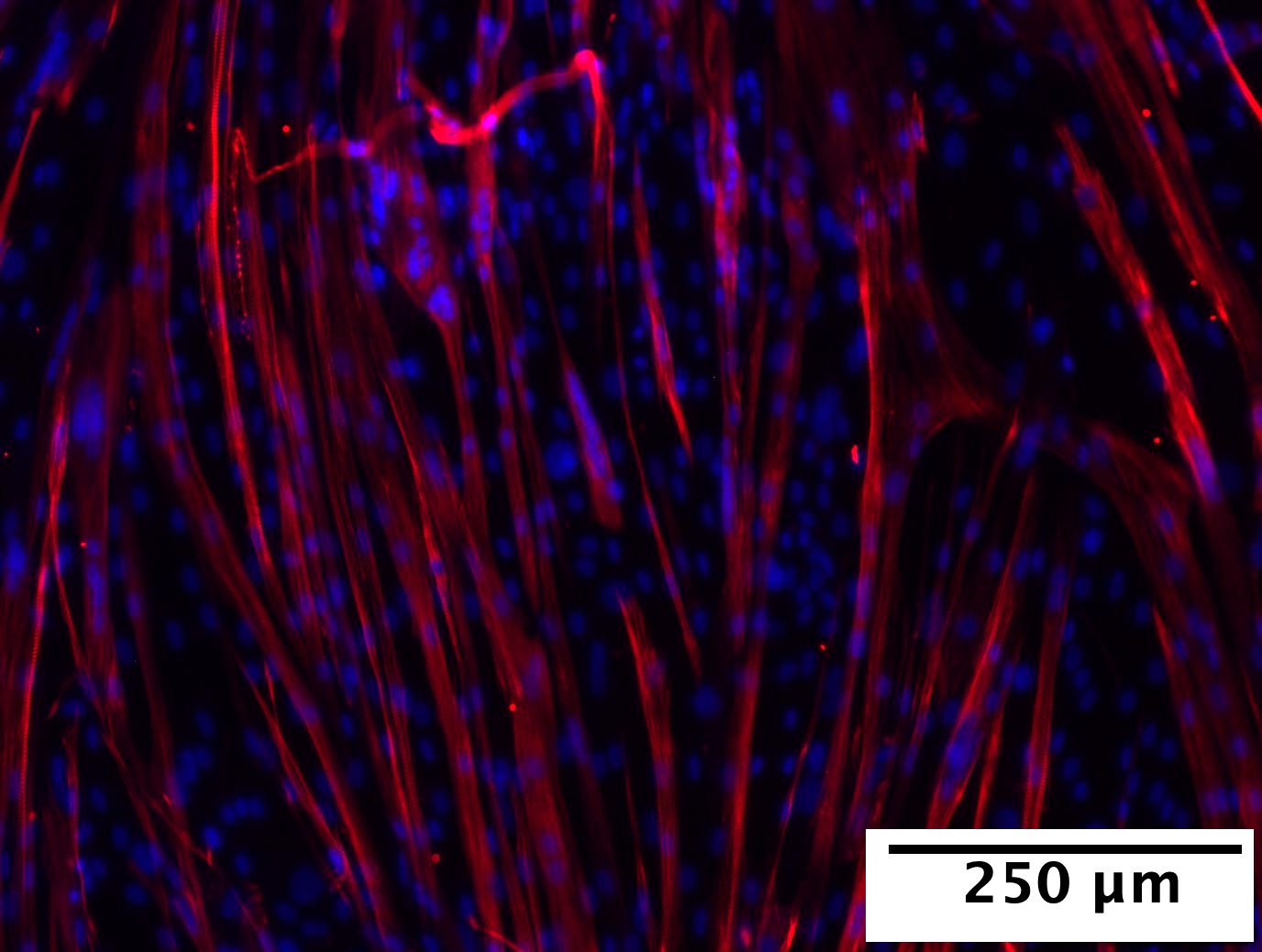


A

F

C

B

E

D

**SF 2:** ICC staining with hMb from gracilis muscle in PP3-P2. blue: cell nuclei (DAPI), red: alpha-actinin and MHC filaments. Upper row (**A-C**): alpha-actinin staining after 3, 7 and 14 days of differentiation, lower row (**D-F**): MHC staining after 3, 7 and 14 days of differentiation. Over time, an increase in myotubes can be seen with increasing incorporation of cell nuclei, so that multinucleated myotubes are formed, as well as a parallel arrangement of the myotubes.

| Examined factors | Study population (n = 18) |
| --- | --- |
|  |  |
| *Gender* |  |
| Male | 6 (33.33 %) |
| Female | 12 (66.67 %) |
|  |  |
| *Age (years)* |  |
| < 50 | 7 (38.89 %) |
| ≥ 50 | 11 (61.11 %) |
|  |  |
| *BMI (kg/m^2^)* |  |
| < 25 | 5 (27.78 %) |
| ≥ 25 | 13 (72.22 %) |
|  |  |
| *Chemotherapy* |  |
| Yes | 7 (38.89 %) |
| No | 11 (61.11 %) |
|  |  |

**SF 3:** Overview of the groups for analysis of the gene expression after differentiation, using a mixed-effect model. Due to the uneven distribution of muscle types and the limited number of irradiated samples, statistical analysis of these factors was not conducted.


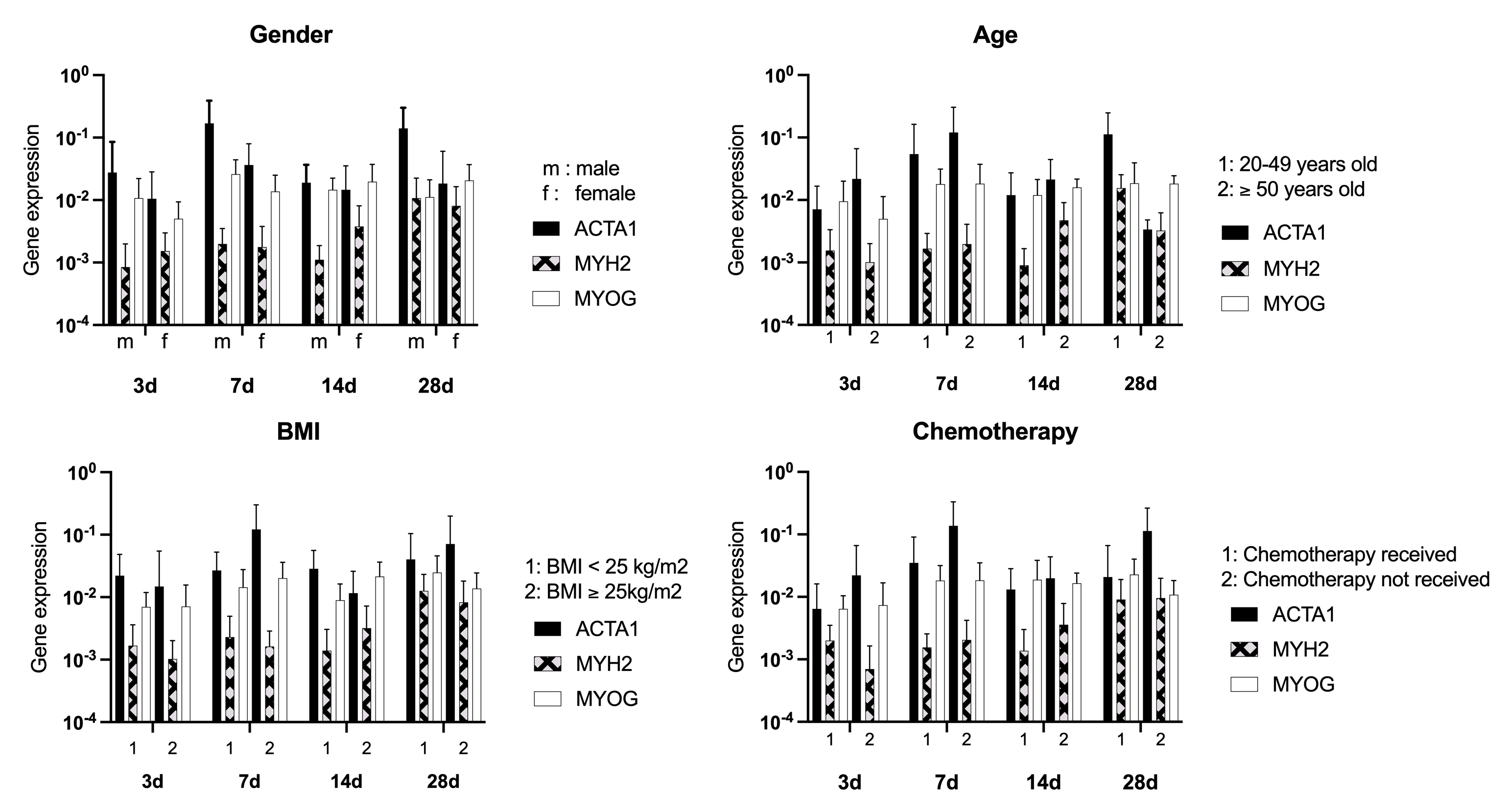


**SF 4:** mRNA expression of ACTA1, MYH2 and MYOG after 3, 7, 14 and 28 days of differentiation. Expression values are shown in $2^{-\Delta CT}$. RPL13a was used as reference gene.
